# Comparative serum cytokine and chemokine profiles across tuberculosis, latent TB, HIV, and COVID-19 infections in Namibian adults

**DOI:** 10.3389/fimmu.2026.1954262

**Published:** 2026-10-02

**Authors:** Ottilie K. H. Katali, David Hertz, Mareli Claassens, Petrina Kapewangolo, Tadesse K. Woldetsadik, Chawangwa Modongo, Stefan Niemann, Bianca E. Schneider, Emmanuel Nepolo

**Affiliations:** 1 Department of Biochemistry, Microbiology and Biotechnology, University of Namibia, Windhoek, Namibia; 2 Department of Human, Biological & Translational Medical Sciences, University of Namibia, Windhoek, Namibia; 3 Host Determinants in Lung Infections, Research Center Borstel, Leibniz Lung Center, Borstel, Germany; 4 Health Poverty Action, London, United Kingdom; 5 Victus Global Botswana Organisation, Gaborone, Botswana; 6 Molecular and Experimental Mycobacteriology, Research Center Borstel, Leibniz Lung Center, Borstel, Germany; 7 German Center for Infection Research (DZIF), Partner Site Hamburg-Lübeck-Borstel-Riems, Borstel, Germany; 8 Phylogenomics, Phylodynamics and Host-Pathogen Interactions team, EPHE, PSL University, Paris, France; 9 Institut de Systématique, Evolution, Biodiversité (ISYEB), MNHN, CNRS, SU, UA, EPHE, Paris, France

**Keywords:** chemokine, COVID-19, cytokine, HIV, infectious disease, inflammatory biomarker, LTBI, TB

## Abstract

**Background:**

The devastating pandemic of coronavirus disease 2019 (COVID-19) highlighted the importance of early detection and global collaboration in combating infectious diseases. Namibia is one of the countries that bears a high burden of tuberculosis (TB) and human immunodeficiency virus (HIV) infection. The overlap of these diseases underscores the need to better understand host immune responses, which play a critical role in disease pathogenesis and may differ across infections. This study aimed to profile selected cytokines and chemokines among Namibian individuals with TB, latent TB infection (LTBI), COVID-19, and HIV infection.

**Methods:**

A cross-sectional study was conducted between August 2022 and May 2023 among adults (≥18 years) recruited from two clinics in Namibia. 222 participants were categorized into multiple clinical groups, including mono- and co-infections of COVID-19, active TB, LTBI, HIV, and control participants. Serum concentrations of 23 cytokines and chemokines were measured using the LEGENDPlex™ multiplex immunoassay. Differences, discriminations and correlations in biomarker levels between groups were analysed using non-parametric statistical tests with adjustment for multiple comparisons. Statistical significance was defined as an adjusted *p* < 0.05.

**Results:**

MCP-1 demonstrated consistent upregulation across active TB, COVID-19, COVID-19/LTBI, and HIV/LTBI groups. In contrast, free active transforming growth factor beta 1 (TGF-β1) was significantly downregulated in both TB and LTBI participants. Individuals with TB exhibited elevated MCP-1 and macrophage migration inhibitory factor (MIF) levels compared to those with LTBI, with both analytes demonstrating discriminatory performance between TB and LTBI. Multiple moderate and weak positive correlations among inflammatory markers were observed in individuals with LTBI and HIV/LTBI but were absent in active TB and COVID-19/LTBI participants.

**Conclusions:**

Our findings demonstrate distinct serum cytokines and chemokines across TB, LTBI, HIV, and COVID-19 in a Namibian cohort. MCP-1 emerged as a consistent marker associated with inflammation. MIF was detected as a potential differentiating marker for TB and LTBI. Larger studies are needed to evaluate the diagnostic potential of these biomarkers and their incorporation into clinical diagnostic algorithms. The results also suggest a possible role for LTBI-associated immune activation in HIV co-infection, which warrants further investigation.

## 1 Introduction

Globally, tuberculosis (TB) caused by pathogens of the *Mycobacterium tuberculosis* complex (Mtbc) and human immunodeficiency virus (HIV) infections continue to pose major public health challenges ranking among the leading causes of morbidity and mortality (1, 2). The 2025 reports of the World Health Organisation (WHO) on TB and United Nations Programmes on HIV/AIDS indicated that about 10.8 million new TB cases were recorded globally, while around 39.9 million people were living with HIV, respectively (1, 3). Namibia bears a particularly heavy burden of both diseases with active TB (referred to as TB in this manuscript) incidence standing at about 468 cases per 100,000 population and approximately 2.2 new HIV infections per 1,000 uninfected individuals by the end of 2025 (4). An estimated quarter of the world’s population carries latent TB infection (LTBI), of whom 5 - 10% will progress to active disease over their lifetime (3). More recently, the global health landscape has also been profoundly affected by the coronavirus disease 2019 (COVID-19) pandemic, caused by Severe Acute Respiratory Syndrome Coronavirus 2 (SARS-CoV-2), which has resulted in over 700 million cases and more than 7 million deaths worldwide as of April 2026 (5).

During the COVID-19 pandemic, COVID-19 and TB adversely affected each other in multiple ways across many countries, including delayed diagnosis and challenges in treatment and clinical management due to their overlapping clinical features (6, 7). Both, COVID-19 and TB primarily affect the lungs and are associated with breathing difficulties, coughing and fever (6, 7). It has been reported that the co-morbidity of pulmonary TB and COVID-19 is likely to prolong respiratory symptoms, fever, and unresolved radiological abnormalities (8, 9) and that patients with LTBI are prone to develop active TB when exposed to SARS-CoV-2 (10). Moreover, emerging evidence suggests that patients with TB, LTBI, HIV or HIV/TB co-infection may be at increased risk of developing severe COVID-19 when infected with SARS-CoV-2, potentially due to impaired or aberrant immune responses during co-infections (11–13).

The dysregulation of the immune system, particularly through excessive cytokine production, plays a central role in the pathogenesis of COVID-19, TB and HIV. This hyperinflammatory state, commonly referred to as a “cytokine storm”, is characterized by an uncontrolled and excessive immune activation resulting in elevated circulating cytokine levels and systemic inflammation (8, 14–18). A cytokine storm is commonly associated with bacterial and viral infections, with varying levels of cytokines reported as inflammatory markers of disease severity in patients infected with COVID-19, TB, or HIV (8, 14–18). Although both COVID-19 and TB, are associated with lung pathologies, they individually have distinct kinetics of cytokine storms (8).

In severe COVID-19, inflammation is characterized by markedly elevated levels of pro-inflammatory mediators including interleukins (IL-6, IL-8, IL-1β), tumor necrosis factor- alpha (TNF-α), and monocyte chemoattractant protein-1 (MCP-1), which are associated with acute respiratory distress syndrome (ARDS), multi-organ failure, and increased mortality (19, 20). Similarly, both TB and LTBI are marked by elevated levels of cytokines such as interferon gamma (IFN-γ), TNF-α, IL-6, IL-10, and IL-17, contributing to immunopathology and lung damage (11, 18, 21). In HIV infection, acute phase immune activation leads to elevated levels of IL-10, IL-18, IFN-γ, and interferon gamma-induced protein 10 (IP-10), although findings remain inconsistent, reflecting complex and dual roles of cytokines in both viral control and immune exhaustion (22–24). Together, these findings underscore the critical role of cytokine networks in determining disease severity across major infectious diseases, highlighting the need for targeted immunomodulatory strategies.

In countries with a high burden of TB, LTBI and HIV, the potential for co-infection with SARS-CoV-2 is substantial, underscoring the need to understand the biological and clinical interactions between these diseases (13). Furthermore, biomarker profiling in TB, LTBI, HIV and COVID-19 have never been investigated among Namibian individuals, despite the high burden of HIV and TB in the country. This analysis was carried out to fill this gap by evaluating and comparing the differences in circulating cytokines and chemokines among Namibian adults with COVID-19, TB, LTBI, and HIV and combinations thereof.

## 2 Materials and methods

### 2.1 Ethics approval

This analysis forms part of the study entitled: Collision of three global pandemics: the effect of tuberculosis and HIV on the epidemiological, clinical, virological and immunological trajectory of COVID-19 in Botswana and Namibia (Core-NB). The study was approved by the University of Namibia (UNAM) Human Research Ethics Committee – Health (Ref No. EXPC/01/21; HREC02/24) and the Ministry of Health and Social Services (MOHSS) (Ref No.17/3/3MC). Written informed consent was received from all study participants. Permission to transfer biological materials from UNAM to the Research Center Borstel (RCB) was granted by MOHSS (Ref No.13/3/2/1).

### 2.2 Participants

Adult participants (≥18 years old) were enrolled from the Epako and Epukiro clinics in Gobabis, Omaheke Region, Namibia, between August 2022 and May 2023. Participants were screened for COVID-19, active TB, LTBI and HIV infection. A standardized questionnaire was administered to participants during sample collection to capture demographic information and clinical characteristics related to the conditions (**Table 1**). Only participants with known COVID-19, HIV, TB, and LTBI statuses were included in the study. The flow chart of the study participant enrolment is shown in **Supplementary Figure 1** Participants- who tested negative for all four conditions in this study were classified as control participants. Participants were not screened for other comorbidities such as hypertension and diabetes; the control group was therefore defined as being free of the four conditions evaluated in this study. Participant enrolment occurred during the period when the Omicron variant of SARS-CoV-2 was predominantly circulating in Southern Africa. Therefore, the COVID-19 participants were presumed to have been infected predominantly with Omicron sub-lineages; however, SARS-CoV-2 lineage confirmation was not performed. At enrolment, 10 participants reported having received at least one dose of a COVID-19 vaccine. Among participants living with HIV, 68.4% (39/57) reported being on antiretroviral therapy (ART). Clinical data including diseases’ severity classifications (mild, moderate, or severe), HIV viral load, CD4 counts, and TB treatment status could not be reliably established at the time of enrolment. None of the participants were hospitalized during enrolment. For additional exploratory analyses, participants with active TB and TB/HIV co-infection were combined into a TB″ group, while participants with COVID-19 mono-infection, COVID-19/HIV co-infection, and COVID-19/LTBI co-infection were combined into a COVID-19″ group.

**Table 1:** Demographics and clinical characteristics of the study participants.

Characteristics	Control participants	LTBI	TB	TB/HIV	TB’’	HIV	HIV/ LTBI	COVID-19	COVID-19/ LTBI	COVID-19/HIV	COVID-19’’	ALL
*n* (%)	54 (24.3)	81 (36.5)	13 (5.9)	4 (1.8)	17 (7.7)	23 (10.4)	28 (12.6)	5 (2.3)	12 (5.4)	2 (0.9)	19 (8.6)	222 (100)
Gender (M/F)	23/31	43/38	6/7	1/3	7/10	12/11	10/18	3/2	3/9	1/1	7/12	102/120
Age (years)	36 [27-457]	31 [24-41]	34 [30-36]	40 [34-44]	34 [30-42]	45 [31-50]	44 [28-52]	27 [22-40]	38 [23-45]	44 [39-48]	34 [23-44]	35 [25-48]
Height (cm)	167 [160-170]	165 [158-171]	155^**^ [153-160]	149 [145-156]	155^**^ [149-160]	165 [155-168]	160 [157-169]	160 [155-165]	161 [158-166]	166 [165-166]	162 [157-166]	165 [157-170]
Weight (kg)	62 [54-69]	55^*^ [49-63]	43^***^ [39-45]	44 [42-50]	44^***^ [39-45]	58 [51-68]	54 [48-67]	52 [46-65]	60 [44-79]	55 [55-55]	55 [46-68]	56 [48-65]
BMI (kg/m^2^)	21.9 [19.7-27.2]	19.8^**^ [17.5-22.0]	17.6^**^ [16.5-19.0]	20.8 [19.4-21.7]	17.9^**^ [16.7-20.4]	21.8 [19.5-25.7]	20.6 [18.9-24.2]	18.0 [17.8-23.9]	22.2 [18.7-30.3]	20.1 [20.0-20.1]	20.2 [17.9-27.8]	20.5 [18.3-24.6]
Smoking: (Yes/No)	6/48	23/57^a^	5/8	1/3	6/11	6/16^a^	6/22	3/2	2/10	2/0	7/12	54/166^a^
Alcohol (Yes/No)	15/38^a^	31/50	6/7	0/4	6/11	11/11^a^	13/15	2/3	3/9	0/2	5/14	81/139*
ART Treatment	–	–	–	4	–	15	19	–	–	1	–	39
COVID-19 Vac (Y/N)	32/22	49/32	2/11	2/2	4/13	18/5	14/14	3/2	7/5	0/2	10/9	141/117
Vac dosage (1^st^/2^nd^/B)	32/10/2	49/23/6	2/2/1	2/0/0	4/2/1	18/15/4	14/12/1	3/2/1	7/2/0	–	10/4/1	141/72/17

Data is presented as Median with interquartile range [IQR] in brackets. TB, tuberculosis; LTBI, latent TB infection; HIV, human immunodeficiency virus; COVID-19, coronavirus disease 2019; *n*, number of participants; BMI, body mass index; ART, antiretroviral therapy; Vac, Vaccination; B, Booster. ^a^Represents the missing data; The double right quotation marks (‘‘) represents the combined groups: TB mono infection with TB/HIV co-infection and COVID-19 mono-infection with COVID-19/HIV and COVID-19/LTBI co-infection, respectively. Statistical comparisons for continuous variables between controls and clinical conditions were performed using the Wilcoxon rank-sum test, with *p*-values adjusted for multiple comparisons using the Holm method. Adjusted *p* values are indicated as follows *p < 0.05; **p < 0.01, *** p < 0.001.

### 2.3 Sample collection and characterization

Participants had rapid HIV testing (CTK Biotech, Inc., Poway, CA, USA) and provided specimens including blood, sputum and nasopharyngeal swabs which, were transported from the clinics to the UNAM Molecular Diagnostic Laboratory within 24 hours. Sputum samples and nasopharyngeal swabs were analyzed for TB using GeneXpert Ultra (Cepheid, Sunnyvale, CA, USA) and COVID-19 infections using qRT-PCR-based SARS-CoV-2 (Wuhan EasyDiagnosis Biomedicine Co., Ltd, Wuhan, China). The QuantiFERON-TB Gold Plus Test (QIAGEN, GmbH, Hilden, Germany) was performed to determine LTBI status. Serum samples were stored at -80 °C until transported on dry ice to RCB in May 2025 for experiments. Prior to experimentation, frozen serum samples were thawed, vortexed, briefly centrifuged to remove particulates, and aliquoted into 96-well plates to minimize repeated freeze-thaw cycles during multiple experimental runs.

### 2.4 Quantification of serum cytokines and chemokines using multiplex immunoassay

Serum concentrations of cytokines and chemokines were measured in duplicates using the LEGENDPlex™ multiplex immunoassay (BioLegend, United Kingdom) according to the manufacturer’s instructions using pre-defined panels. The 23 chemokines and cytokines measured were IL-1β, IL-1RA, IL-2, IL-3, IL-4, IL-6, IL-8, IL-10, CXCL10 (IP-10), IL-13, TNF-α, CCL2 (MCP-1), IL-17A, IFN-γ, IL-12p70, Free active TGF-β1, CCL5 (RANTES), NCAM, IFN-α2, MIF, sCD14, sTREM-1 and SAA. Briefly, 25 µL of Matrix B and 25 µL of assay buffer were added to the standards and sample wells, respectively, of a V-bottom plate. Subsequently, 25 µL of vortexed mixed beads were added to each well followed by 25 µL of thawed undiluted serum samples, reconstituted standards, and controls. The plate was incubated for 2 hours at room temperature (RT) on a shaker (800 rpm). Following incubation, the bead pellets were washed twice with 200 µL of 1× wash buffer. Next, 25 µL of detection antibodies were added to each well and incubated for 1 hour at RT. Without washing, 25 µL of streptavidin-phycoerythrin (SA-PE) was added and incubated for 30 minutes at RT. The beads were then centrifuged and resuspended in 150 µL of 1× wash buffer prior to acquisition. The plate was read on a flow cytometer (MACSQuant Analyzer 10, Miltenyi Biotec B.V. & Co. KG, Germany). All incubation steps were performed at room temperature on a shaker (800 rpm) with plates covered with foil. Standards, controls and samples were run in duplicate. Analyte concentrations were calculated from standard curves generated using the mean fluorescence intensity (MFI) of standards with known concentrations and expressed in pg/mL.

### 2.5 Statistical analysis

The raw data files obtained from the assays were organized and cleaned in batches then combined in one excel data file. Analyte concentration values that fell below or above the assay detection limits were imputed as 10% lower than the minimum or 10% higher than the maximum concentration for each analyte, in accordance with the approach described by Kleynhans et al. (25). Statistical analyses were performed using R (version 4.5.2). Data distribution was assessed using skewness and histogram inspection and was found to be non-normal. Non-parametric methods were applied for group comparisons. Analytes values were winsorized at the 5th and 95th percentiles across the entire cohort prior to analysis to reduce the impact of outliers. Data are presented as median (interquartile range, IQR). Both raw and adjusted p-values are reported in **Table 2** and in the **Supplementary Tables** to allow clear interpretation of comparisons that did not remain significant after correction for multiple testing. Group comparisons were performed using the Wilcoxon rank-sum test, while correlations were assessed using Spearman’s rank correlation test. Receiver operating characteristic (ROC) curve analyses and area under the curve (AUC) calculations were performed to evaluate discriminatory performance of selected analytes between groups. Correction for multiple testing was performed using the Benjamini–Hochberg method for cytokine and chemokine analyses to control the false discovery rate, and the Holm method for clinical and demographic comparisons to control the family-wise error rate. Statistical significance was set at an adjusted *p*-value < 0.05. Correlation strength for spearman rho was defined as strong ≥ 0.70, moderate (M), 0.40-0.69, weak (W), 0.20-0.39. ROC performance was interpreted according to the AUC as follows: poor (<0.60), weak (0.60–0.69), acceptable (0.70–0.79), good (0.80–0.89), and excellent (≥0.90).

**Table 2:** Significant correlations between serum levels of cyto- and chemokines in individuals with LTBI and HIV/LTBI.

Condition	Correlated analytes	Spearman’s rho (ρ)	raw *p* value	adj. *p* value	Strength
LTBI	MIF vs IL-1RA	0.67	7.96×10^-12^	1.67×10^-10^	Moderate
LTBI	IL-8 vs IL-12p70	0.42	1.01×10^-4^	0.00106	Moderate
LTBI	MCP-1 vs MIF	0.33	0.00258	0.0145	Weak
LTBI	IL-8 vs Free active TGF-β1	0.33	0.00275	0.0145	Weak
LTBI	IL-1RA vs Free active TGF-β1	0.31	0.00513	0.0216	Weak
LTBI	MIF vs IL-12p70	0.27	0.014	0.049	Weak
HIV/LTBI	IL-1RA vs Free active TGF-β1	0.57	0.00164	0.0247	Moderate
HIV/LTBI	IP-10 vs IL-1RA	0.51	0.00506	0.038	Moderate
HIV/LTBI	IL-8 vs IL-1RA	0.49	0.00859	0.043	Moderate
HIV/LTBI	MCP-1 vs IL-1RA	0.46	0.013	0.0461	Moderate
HIV/LTBI	IP-10 vs Free active TGF-β1	0.45	0.0154	0.0461	Moderate

TB, tuberculosis; LTBI, latent TB infection; HIV, human immunodeficiency virus; IL, interleukin; MCP-1, monocyte chemoattractant protein-1; MIF, macrophage migration inhibitory factor; IP-10, interferon gamma-induced protein 10; vs, Versus. Correlation between the analytes were performed using the Spearman’s rank correlation test, with p-values adjusted for multiple comparisons using the Benjamini–Hochberg method. Statistical significance was defined as adjusted (adj.) p < 0.05. Correlation strength for spearman rho was defined as strong ≥ 0.70, moderate (M), 0.40-0.69, weak (W), 0.20-0.39.

## 3 Results

### 3.1 Participant demographics and clinical characteristics

Two hundred and twenty-two participants were enrolled in this study. Of these, 81 (36.5%), 13 (5.9%), 23 (10.4%) and 5 (2.3%) had LTBI-, TB-, HIV- and COVID-19, respectively. Those who had more than one condition were 28 (12.6%) with HIV/LTBI, 12 (5.4%) with COVID-19/LTBI, four (1.8%) with TB/HIV and two (0.9%) with COVID-19/HIV. The control participants made up 24.3% (54) of the participants. Demographics and clinical characteristics are shown in **Table 1**. The median age [IQR] of the participants was 35 years [25-48], which did not differ significantly between the groups. There were significant differences between the weight (adj. *p* = 0.002), height (adj. *p* < 0.001) and BMI (adj. *p* = 0.003) of the TB participants and the control participants. There were also significant differences between the weight (adj. *p* = 0.033) and BMI (adj. *p* = 0.009) of the LTBI participants and the control participants. 24.3% and 36.4% of the total participants reported smoking and drinking alcohol at the time of recruitment.

### 3.2 Quantification of serum cytokines and chemokines

Twenty-three cytokines and chemokines were measured in the serum samples of the participants. Of these, ten cytokines (IL-1β, IL-2, IL-3, IL-4, IL-6, IL-13, TNF-α, IL-17A, IFN-γ and IFN-α2) were below the detection limit and could not be quantified, while three (CCL5, sCD14 and SAA) were above the detection limit. Cytokines and chemokines within the measurable range were IL-1RA, IL-8, IL-10, CXCL10 (IP-10), CCL2 (MCP-1), IL-12p70, free active TGF-β1, NCAM, MIF, and sTREM-1 (**Figures 1**–**4**). These analytes serum concentrations are shown in **Supplementary Table 1**.

**Figure 1:**
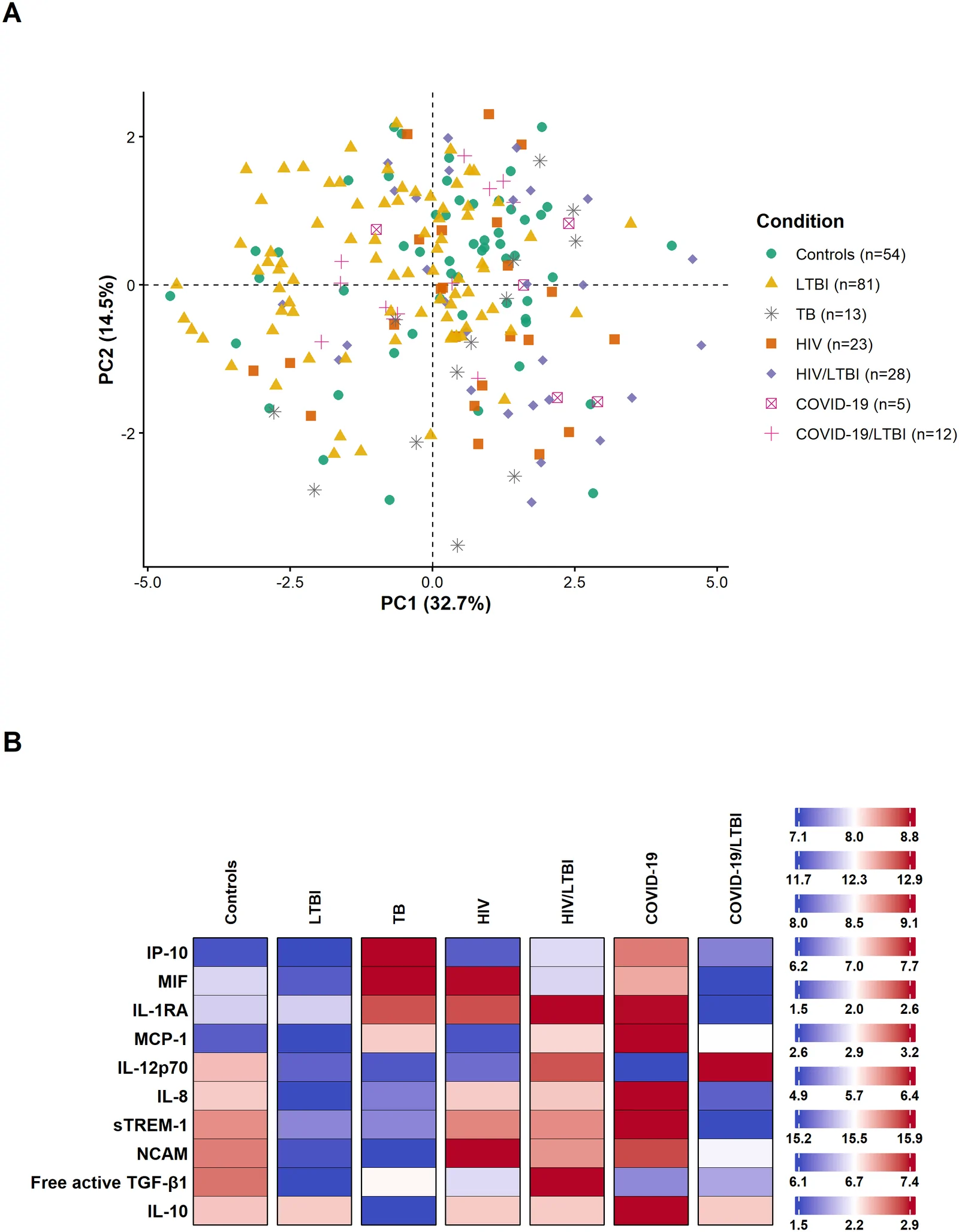
Variation and patterns among cytokine and chemokine (IL-1RA, IL-8, IL-10, CXCL10 (IP-10), CCL2 (MCP-1), IL-12p70, free active TGF-β1, NCAM, MIF, and sTREM-1 expression across clinical conditions: control participants (*n* = 54), LTBI (*n* = 81), TB (*n* = 13), COVID-19 (*n* = 5), COVID-19/LTBI (*n* = 12), HIV (*n* = 23) and HIV/LTBI (*n* = 28). **(A)** PCA of cytokine and chemokine expression across clinical conditions. Each point represents an individual participant with ellipses representing the distribution of each clinical group in the PCA space. **(B)** Heatmap showing the relative distribution of 10 cytokines and chemokines measured across study participants with different clinical conditions: Values were first winsorized at the 5th and 95th percentiles to reduce the influence of extreme values then log2(x+1)-transformed to reduce skewness and improve visualization across analytes with differing concentration ranges. Colour intensity represents relative analyte levels (blue = low, white = intermediate, red = high). Each column represents the median of the condition, and each row represents a cytokine or chemokine. Samples were ordered by clinical group and were not clustered.

**Figure 2:**
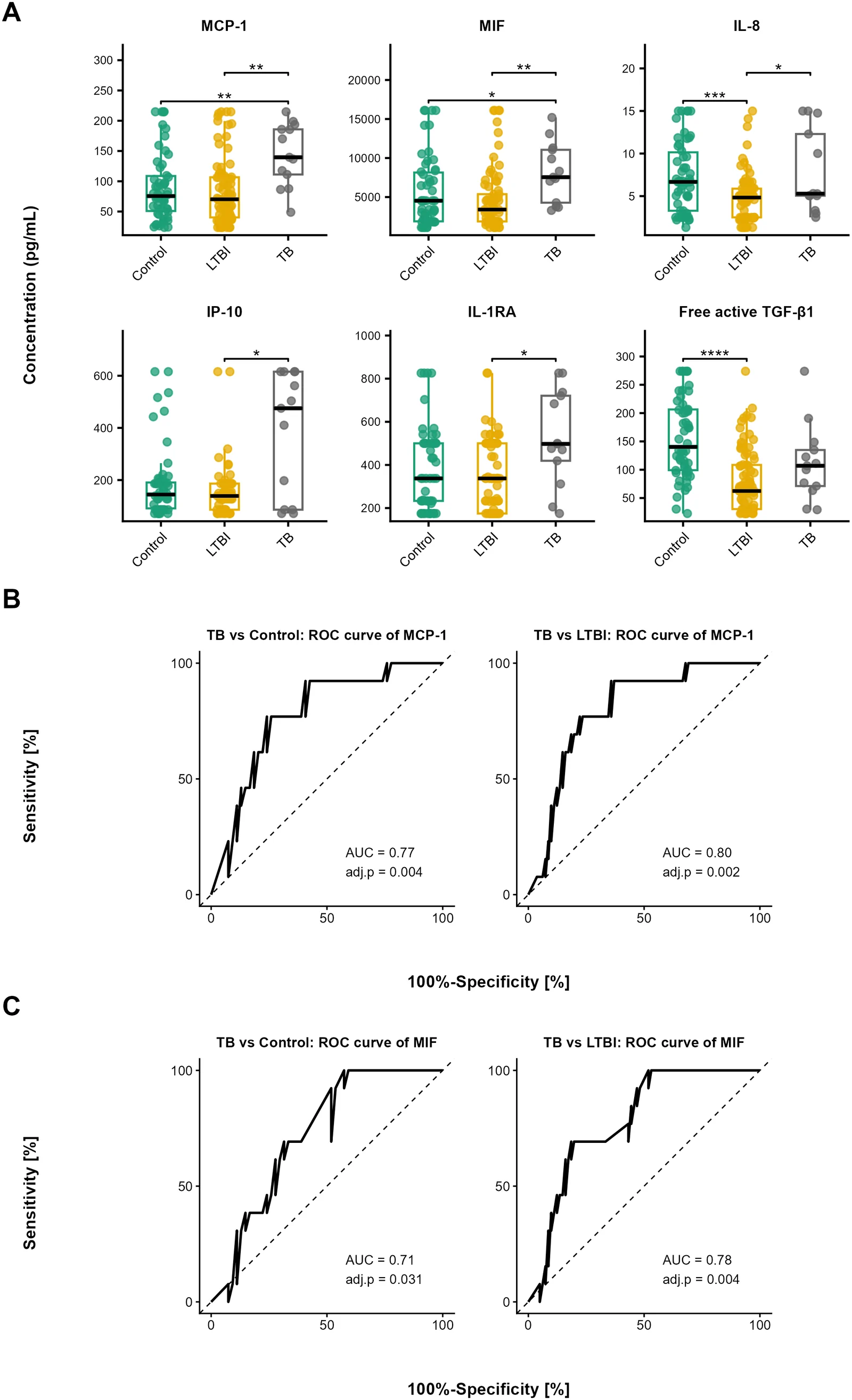
Serum concentrations of MCP-1, MIF, IL-8, IP-10, free active TGF-β1, and IL-1RA among control participants (Control, *n* = 54), individuals with tuberculosis (TB, *n* = 13), and those with latent tuberculosis infection (LTBI, *n* = 81). **(A)** Comparison of cytokine and chemokine concentrations between the groups. **(B, C)** Discriminatory performance of MCP-1 and MIF between the groups assessed using receiver operating characteristic (ROC) curves and area under the curve (AUC) analysis. To reduce the influence of extreme values, data were winsorized at the 5th and 95th percentiles prior to analysis. Statistical comparisons between groups were performed using the Wilcoxon rank-sum test, with p-values adjusted for multiple comparisons using the Benjamini–Hochberg method. Data are presented as box plots showing the median (line) and interquartile range (box), with each point representing an individual participant. Statistical significance was defined as adjusted p < 0.05 and is indicated as follows: *p < 0.05; **p < 0.01; ***p < 0.001; ****p < 0.0001.

**Figure 3:**
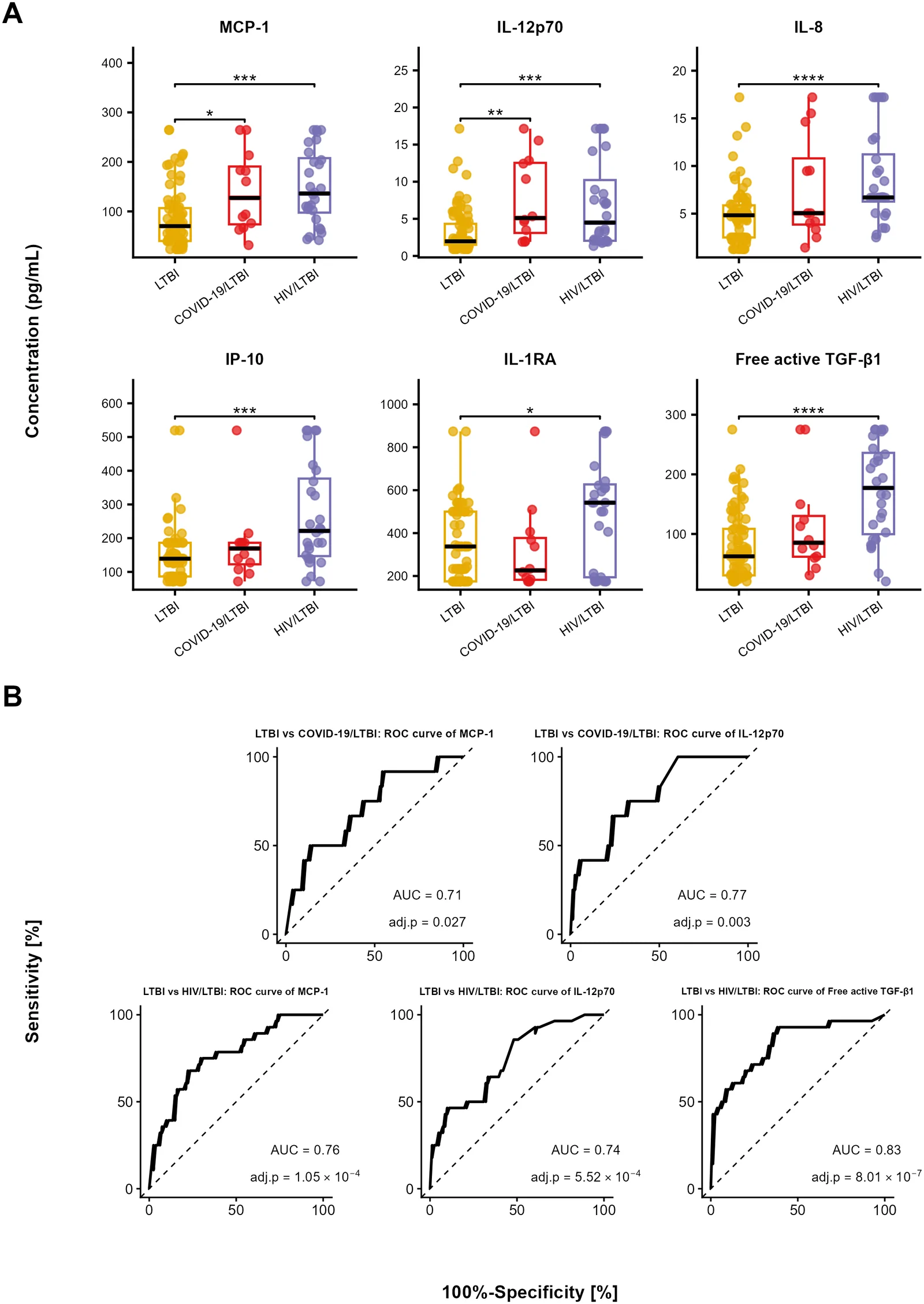
Serum concentrations of MCP-1, IL-12p70, IL-8, IP-10, free active TGF-β1, and IL-1RA among individuals with LTBI (n = 81), COVID-19/LTBI (n = 12), and HIV/LTBI (n = 28). **(A)** Comparison of cytokine and chemokine concentrations between the groups. **(B)** Discriminatory performance of MCP-1, MIF and free active TGF-β1 between the groups assessed using receiver operating characteristic (ROC) curves and area under the curve (AUC) analysis. To reduce the influence of extreme values, data were winsorized at the 5th and 95th percentiles prior to analysis. Statistical comparisons between groups were performed using the Wilcoxon rank-sum test, with p-values adjusted for multiple comparisons using the Benjamini–Hochberg method. In **(A)**, data are presented as box plots showing the median (line) and interquartile range (box), with each point representing an individual participant. Statistical significance was defined as adjusted p < 0.05 and is indicated as follows: *p < 0.05; **p < 0.01; ***p < 0.001; ****p < 0.0001.

**Figure 4:**
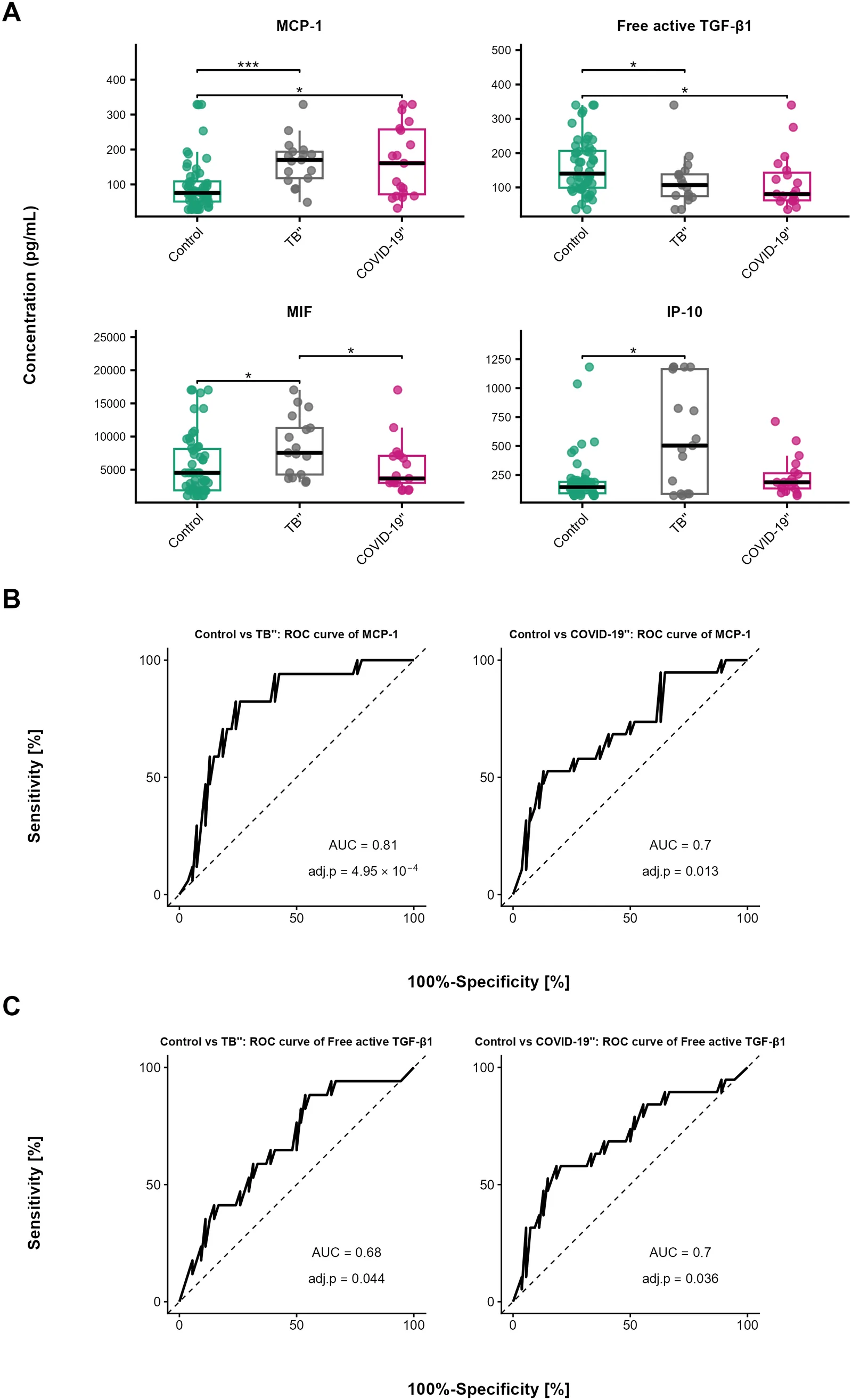
Serum concentrations of MCP-1, free active TGF-β1, MIF and IP-10 among control participants (*n* = 54), individuals with tuberculosis (*n* = 17), and those with COVID-19 infection (*n* = 19). The double right quotation marks (‘‘) represents the combined groups: TB with TB/HIV and COVID-19/HIV, COVID-19/LTBI with COVID-19, respectively. **(A)** Comparison of cytokine and chemokine concentrations between the groups. **(B, C)** Discriminatory performance of MCP-1 and free active TGF-β1 between the groups assessed using receiver operating characteristic (ROC) curves and area under the curve (AUC) analysis. To reduce the influence of extreme values, data were winsorized at the 5th and 95th percentiles prior to analysis. Statistical comparisons between groups were performed using the Wilcoxon rank-sum test, with p-values adjusted for multiple comparisons using the Benjamini–Hochberg method. Data are presented as box plots showing the median (line) and interquartile range (box), with each point representing an individual participant. Statistical significance was defined as adjusted *p* < 0.05 and is indicated as follows: *p < 0.05; ***p < 0.001.

### 3.3 Variation and patterns among cytokine and chemokine expression across clinical conditions

An unsupervised principal component analysis (PCA) was done to visualize variation in cytokine and chemokine profiles across clinical conditions (**Figure 1A**). The PCA did not reveal clear clustering by disease group, indicating that the global cytokine variation is dominated by inter-individual heterogeneity rather than disease-specific effects. However, examination of individual cytokines and chemokines using a heatmap revealed differences between disease groups. (**Figure 1B**). Values were normalized per analyte using its empirical distribution (5th percentile, median, and 95th percentile) to enable comparison of relative expression patterns across cytokines with different dynamic ranges.

### 3.4 Serum levels of inflammatory markers in participants with TB and LTBI infections

To determine the impact of LTBI and TB on serum cytokine and chemokine levels, these two groups were compared to the control participants as well as to each other. Six out of 23 cytokines and chemokines tested showed statistically significant difference (**Figure 2A**). TB participants showed significantly higher levels of MCP-1 (adj. *p* = 0.004) and MIF (adj. *p* = 0.031) compared to control participants. In addition, MCP-1 (adj. *p* = 0.002), MIF (adj. *p* = 0.004), IL-8 (adj. *p* = 0.028), IP-10 (adj. *p* = 0.042) and IL-1RA (adj. *p* = 0.029) were significantly higher in individuals with TB compared to LTBI. ROC curve analyses and AUC calculations were performed to evaluate discriminatory performance of selected analytes between groups. MCP-1 and MIF demonstrated acceptable to good discriminatory performance in distinguishing TB from both LTBI and control participants (**Figures 2B, C**, **Supplementary Table 2**). Furthermore, individuals with LTBI had significantly lower levels of free active TGF-β1 levels and IL-8 (adj. *p* = 3.67 × 10^-4^) compared to control participants (adj. *p* = 3.62 × 10^-9^).

We next explored the impact of seropositive SARS-CoV-2 or HIV on baseline serum cyto- and chemokine levels in individuals with LTBI. As shown in **Figure 3A**, there were significant increased levels of MCP-1 in both individuals with COVID-19/LTBI (adj. *p* = 0.027) and HIV/LTBI (adj. *p* = 1.05 × 10^-4^) compared to those with LTBI (**Figure 3A**). Similarly, IL-12p70 was elevated in individuals with COVID-19/LTBI (adj. *p* = 0.003) and HIV/LTBI (adj. *p* = 5.53 × 10^-4^) compared to LTBI individuals. In contrast, significant elevation of IL-8 (adj. *p* = 9.48×10^-6^), IP-10 (adj. *p* = 0.0002), IL-1RA (adj. *p* = 0.012) and free active TGF-β1(adj. *p* = 8.01×10^-7^) were only observed in individuals with HIV/LTBI but not in those with COVID-19/LTBI when compared to individuals with LTBI only. Of these, free active TGF-β1 showed good discriminatory power to discriminate between LTBI and HIV/LTBI, with an AUC (95%CI) of 0.827 (0.733-0.922). MCP-1 effectively distinguished LTBI from COVID-19/LTBI and from HIV/LTBI, while IL-12p70 showed similar discriminatory power between LTBI and COVID-19/LTBI (adj. *p* = 0.003) and between LTBI and HIV/LTBI (**Figure 3B**, **Supplementary Table 3**).

### 3.5 Serum levels of inflammatory markers in participants with TB vs COVID-19 infections

For exploratory analyses to assess biomarker levels associated with active TB and COVID-19, serum cytokine and chemokine concentrations were analyzed between these groups, regardless of HIV and LTBI status, and compared with control participants (**Figure 4A**). MCP-1 was significantly upregulated in both TB (adj. *p* = 4.96×10^-4^) and COVID-19 (adj. *p* = 0.013) participants compared to control participants. In contrast, free active TGF-β1 was significantly downregulated in both TB (adj. *p* = 0.044) and COVID-19 (adj. *p* = 0.036) participants. Individuals with TB also exhibited significantly elevated levels of IP-10 (adj. *p* = 0.032) and MIF (adj. *p* = 0.015) compared to control participants. Furthermore, COVID-19 participants showed significantly lower levels of MIF (adj. *p* = 0.015) compared to TB patients. ROC curve analyses and AUC calculations revealed good to acceptable discriminatory power for MCP-1 to discriminate between control participants and TB patients as well as between control participants and individuals with COVID-19 whereas free active TGF-β1 showed weak discriminatory performance in these groups (**Figure 4B, C**, **Supplementary Table 4**).

### 3.6 Correlation analysis of serum cytokines in individuals with TB, LTBI, COVID-19/LTBI and HIV/LTBI

A correlation analysis was performed among analytes that demonstrated significant differences across the clinical group comparisons (**Table 2**, **Supplementary Table 5**). Multiple moderate and weak positive correlations were observed among analytes in LTBI and HIV/LTBI individuals (**Table 2**). In contrast, no significant correlations were detected in TB, or COVID-19/LTBI participants (**Supplementary Table 5**).

## 4 Discussion

TB, LTBI, COVID-19, and HIV are associated with the release of cytokines and chemokines, which play an important role in host immune defence (8, 17, 26). Cytokines and chemokines have emerged as promising biomarkers and biosignatures for the diagnosis, monitoring, and prognosis of infectious diseases (27). In this analysis, we investigated the immune profiles of TB, LTBI, COVID-19, and HIV infection in Namibian individuals. The study provides new insights in immune profile of people with TB, LTBI, COVID-19, and HIV infection, and contributes novel data from Namibia, a setting with limited published immunological biomarker profiling studies.

Our results demonstrated a significant higher serum level of MCP-1(CCL2) in individuals with TB and COVID-19 infections. Higher levels of MCP-1 have been previously reported in TB (11) and COVID-19 patients, with some studies associating it with severe conditions and fatalities (12, 28–31). The observation of elevated MCP-1 levels in both TB and COVID-19 suggests that MCP-1 may represent a common biomarker of infection-induced myeloid inflammation rather than a disease-specific signature. MCP-1 plays an important role in the mechanism of recruitment and activation of inflammatory cells such as macrophages and monocytes at the site of inflammation and has been associated with various pathogenesis of many diseases including TB and COVID-19 (32, 33).

In addition, MCP-1 levels were significantly elevated in individuals with TB compared to those with LTBI, which discriminated between the two conditions. These findings are consistent with previous reports, including that of Luo and colleagues (34), which identified MCP-1 as a potential biomarker capable of differentiating between TB, LTBI, and healthy individuals in a Chinese adult population. In agreement with these observations, individuals who progressed from LTBI to active TB have also been reported to exhibit elevated MCP-1 levels alongside other key chemokines (34). Our findings underscore the potential of MCP-1 as a biomarker for differentiating LTBI from active TB, thereby showing promising discriminatory potential in this exploratory cohort. Notably, our results further demonstrated that MCP-1 levels were significantly higher in individuals with HIV/LTBI co-infection. Elevated MCP-1 levels in individuals with HIV/LTBI may reflect an inflammatory milieu associated with increased susceptibility to TB reactivation. This interpretation is consistent with studies linking elevated MCP-1 expression to progression from latent to active TB, as well as with the well-established increased risk of TB reactivation in people living with HIV.

The pro-inflammatory cytokine IL-12p70 was reported previously to be a potential discriminant between LTBI and TB in a Ghanaian cohort (35). However, our study did not observe this discrimination between LTBI and TB. In contrast, IL-12p70 differentiated LTBI from the groups COVID-19/LTBI and HIV/LTBI. Similarly, elevated levels of IL-12p70 in individuals with HIV/LTBI individuals but not in those with LTBI only were observed in a Kenyan cohort (36). These findings may suggest that immune regulatory pathways associated with LTBI infection could be altered by other conditions such as HIV infection and COVID-19.

Our study further demonstrated IP-10 to be significantly elevated in TB compared to LTBI, which supports previous observations of this marker differentiating between the two conditions (37–39). IP-10 levels were also significantly higher in HIV/LTBI co-infection compared to LTBI individuals. In contrast to MCP-1, IP-10 was not increased in individuals with COVID-19. Another pro-inflammatory cytokine that was highlighted in our analysis was MIF, which showed higher levels in individuals with TB when compared to control participants, LTBI, and COVID-19 participants. This cytokine was previously reported to have potential as a prognostic marker for TB patients. Shang et al. reported it to exhibit higher serum levels in individuals with TB at baseline, which decrease with anti-TB treatment to near normal (40). Furthermore, higher serum levels of MIF were also determined by Wang et al. to be a good indicator of higher risk of severity and mortality among TB patients (41). In contrast to our study, MIF was previously reported to be higher in COVID-19 patients when compared to the control group (42) and associated with severity (43, 44).

In contrast to the pro-inflammatory mediators that were found to be elevated in TB patients, free active TGF-β1 levels were reduced in individuals with TB, LTBI, or COVID-19. TGF-β1 predominantly circulates in a latent inactive form and requires activation before becoming biologically active and immunoreactive. Hence, while active TGF-β1 represents the biologically available fraction capable of mediating downstream signaling, it does not reflect overall cytokine abundance. Previous studies in COVID-19 have reported conflicting findings regarding TGF-β1. For example, reduced inhibitory effects of TGF-β1 in severely ill COVID-19 patients have been suggested to contribute to enhanced pro-inflammatory activity (45). In contrast, Frischbutter et al. reported that higher TGF-β1 levels were associated with increased disease severity in COVID-19 patients (46). Notably, these previous studies measured total TGF-β1 following activation prior to analysis. Therefore, in our analysis, total TGF-β1 may have been present but remained in its latent or bound form rather than as freely active TGF-β1.

Our study demonstrated several moderate to weak positive correlations among immune mediators in individuals with LTBI and HIV/LTBI participants, whereas no correlations were observed in individuals with TB, and COVID-19/LTBI participants. However, absent of correlations in TB and COVID-19/LTBI groups could mostly be due to small sample sizes of these groups compared to the LTBI and HIV/LTBI groups. Several chemokines and cytokines including MCP-1, MIF, and interleukins are known to play important roles in protective immunity including innate and adaptive immune responses (47–49). MCP-1 is mainly involved in monocyte and macrophage recruitment (48), while MIF stimulates macrophage activation (47). In our analysis, IL-1RA demonstrated positive correlations with the pro-inflammatory markers IP-10, IL-8, and MCP-1 in individuals with HIV/LTBI, as well as with MIF in individuals with LTBI. These findings may suggest coordinated activation of inflammatory and compensatory regulatory immune pathways during LTBI and HIV/LTBI co-infection. Furthermore, reduced serum IL-1RA levels have previously been associated with LTBI treatment (50), suggesting a potential role for IL-1RA in LTBI immune regulation.

Although COVID-19 and TB are mainly characterized by pulmonary pathology, both conditions have been reported to exhibit distinct cytokine storm kinetics (8). It is also reported that ethnicity plays a role in the expression of cytokines and chemokines (26). Consequently, the considerable variability in cytokine and chemokine expression patterns reported across studies complicates the interpretation of their precise roles in infectious disease pathogenesis. In our study, comparisons between TB and COVID-19 were performed using pooled groups regardless of HIV and/or LTBI status. Therefore, HIV and LTBI status may have acted as potential confounding factors influencing the observed biomarker profiles. Findings from these pooled analyses should therefore be interpreted with caution and regarded as exploratory rather than confirmatory.

Several limitations should be considered when interpreting our findings. Firstly, some subgroups had relatively small sample sizes, which may have reduced statistical power and may also increase variability, which affects the ability to generalize the findings. Nevertheless, to reduce the likelihood of false-positive results arising from multiple comparisons, adjusted p-values were applied during statistical analysis. However, such adjustments can be conservative, particularly in analyses involving small sample sizes, and may increase the likelihood of false-negative findings. Therefore, raw *p*-values are also reported alongside adjusted *p*-values to provide transparency and facilitate cautious interpretation, particularly for analytes that were significant based on raw *p*-values but did not remain significant after multiple-testing correction. Secondly, the cross-sectional design of the study limits the ability to establish changes in cytokine and chemokine levels during disease progression. Thirdly, differences in disease-related, demographic, and lifestyle factors may have contributed to variability in the observed cytokine and chemokine profiles. Although data on age, sex, BMI, smoking, alcohol use, and ART status were available, these variables were not adjusted for in the biomarker analyses because of the small sample sizes within some clinical subgroups. In addition, information on disease severity, CD4 counts, HIV viral load, and TB treatment status was not available for this analysis. Therefore, the potential influence of these factors on the observed biomarker differences cannot be excluded and should be considered when interpreting the findings. Fourthly, cytokine and chemokine measurements were performed using unstimulated serum samples, which may partly explain why several cytokines fell below the detection limit. Many cytokines, including IL-1β, IL-2, IL-4, IFN-γ, TNF-α, and IL-17A, are associated with acute T-cell or inflammatory activation and are therefore expected to occur at very low circulating concentrations. Therefore, the high proportion of values below the detection limits may reflect the low circulating concentrations of these cytokines in unstimulated serum samples. Furthermore, serum cytokines concentrations may not fully reflect cytokine production by peripheral blood cells (51) and stimulated samples may have provided additional information on inducible cytokine responses. The assays were also performed on stored serum samples approximately two years after sample collection rather than on fresh samples. Although the samples were stored at −80 °C, prolonged storage may have influenced the stability and measured concentrations of some analytes and should therefore be considered when interpreting the findings. Lastly, there was no independent validation cohort; therefore, our study findings should be regarded as exploratory rather than confirmatory. Considering the abovementioned limitations, although our study provides preliminary insights into the Namibian population, the findings highlights the need for more detailed investigations in larger cohorts, with a focused evaluation of consistently emerging biomarkers such as CCL2 (MCP-1) and CXCL10 (IP-10), to improve our understanding of their diagnostic and prognostic significance. Further investigation also needs to look into cytokines with contradictory findings across studies, such as TGF-β1 and MIF, to establish a better understanding into their regulations and roles across different infectious diseases.

In conclusion, our findings provide evidence to support that MCP-1 may serve as a marker of disease-associated inflammation across TB, COVID-19, and LTBI-associated infections. MIF showed potential utility as differentiating markers between TB and LTBI.

We recommend studies with larger cohorts to evaluate whether these biomarkers or their combinations can be incorporated into clinically useful diagnostic models. The results also suggest a possible role for LTBI-associated immune activation in HIV co-infection, which warrants further investigation.

## Data Availability

The original contributions presented in the study are included in the article/**Supplementary Material**. Further inquiries can be directed to the corresponding author.

## References

[1] United Nations Programme On HIV/AIDS . Unaids Epidemiological Estimates (2025). Available online at: https://aidsinfo.unaids.org/ (Accessed April 10, 2026).

[2] World Health Organization . Global Tuberculosis Report 2025. Geneva: World health Organistation (2025). Available online at: https://www.who.int/teams/global-programme-on-tuberculosis-and-lung-health/tb-reports/global-tuberculosis-report-2025 (Accessed April 28, 2026).

[3] World Health Organisation . Tuberculosis (2025). Available online at: https://www.who.int/health-topics/tuberculosis#tab=tab_1 (Accessed April 28, 2026).

[4] World Health Organisation . World Health Statistics 2025 (2025). Available online at: https://www.who.int/data/gho/whs-annex (Accessed April 28, 2026).

[5] Word Health Organisation . Coronavirus Disease (COVID-19) (2025). Available online at: https://www.who.int/emergencies/diseases/novel-coronavirus-2019 (Accessed April 13, 2026).

[6] Kant S Tyagi R . The impact of COVID-19 on tuberculosis: challenges and opportunities. Ther Adv Infect Dis. (2021) 8:1–7. doi: 10.1177/20499361211016973 34178322 PMC8193657

[7] Udwadia ZF Vora A Tripathi AR Malu KN Lange C Sara Raju R . COVID-19 -Tuberculosis interactions: When dark forces collide. Indian J Tuberc. (2020) 67:S155–62. doi: 10.1016/j.ijtb.2020.07.003 33308662 PMC7362784

[8] Udoakang AJ Djomkam Zune AL Tapela K Nganyewo NN Olisaka FN Anyigba CA . The COVID-19, tuberculosis and HIV/AIDS: Ménage à Trois. Front Immunol. (2023) 14:1104828. doi: 10.3389/fimmu.2023.1104828 36776887 PMC9911459

[9] Etna MP Giacomini E Severa M Coccia EM . Pro- and anti-inflammatory cytokines in tuberculosis: A two-edged sword in TB pathogenesis. Semin Immunol. (2014) 26:543–51. doi: 10.1016/j.smim.2014.09.011 25453229

[10] Rajamanickam A Pavan Kumar N Chandrasekaran P Nancy A Bhavani PK Selvaraj N . Effect of SARS-CoV-2 seropositivity on antigen – specific cytokine and chemokine responses in latent tuberculosis. Cytokine. (2022) 150:155785. doi: 10.1016/j.cyto.2021.155785 34933240 PMC8668379

[11] Rajamanickam A Ann Daniel E Dasan B Thiruvengadam K Chandrasekaran P Gaikwad S . Plasma immune biomarkers predictive of progression to active tuberculosis in household contacts of patients with tuberculosis. J Infect Dis. (2025) 231:696–705. doi: 10.1093/infdis/jiae365 39028003 PMC11911786

[12] Chen Y Wang J Liu C Su L Zhang D Fan J . IP-10 and MCP-1 as biomarkers associated with disease severity of COVID-19. Mol Med. (2020) 26:97. doi: 10.1186/s10020-020-00230-x 33121429 PMC7594996

[13] Tamuzi JL Ayele BT Shumba CS Adetokunboh OO Uwimana-Nicol J Haile ZT . Implications of COVID-19 in high burden countries for HIV/TB: A systematic review of evidence. BMC Infect Dis. (2020) 20:744. doi: 10.1186/s12879-020-05450-4 33036570 PMC7545798

[14] Fajgenbaum DC June CH . Cytokine storm. N Engl J Med. (2020) 383:2255–73. doi: 10.1056/NEJMra2026131 33264547 PMC7727315

[15] Gu Y Hsu ACY Pang Z Pan H Zuo X Wang G . Role of the innate cytokine storm induced by the influenza A virus. Viral Immunol. (2019) 32:244–51. doi: 10.1089/vim.2019.0032 31188076

[16] Montazersaheb S Hosseiniyan Khatibi SM Hejazi MS Tarhriz V Farjami A Ghasemian Sorbeni F . COVID-19 infection: an overview on cytokine storm and related interventions. Virol J. (2022) 19:92. doi: 10.1186/s12985-022-01814-1 35619180 PMC9134144

[17] Teijaro JR . Cytokine storms in infectious diseases. Semin Immunopathol. (2017) 39:501–3. doi: 10.1007/s00281-017-0640-2 28674818 PMC7079934

[18] Vivekanandan MM Adankwah E Aniagyei W Acheampong I Yeboah A Arthur JF . Plasma cytokine levels characterize disease pathogenesis and treatment response in tuberculosis patients. Infection. (2023) 51:169–79. doi: 10.1007/s15010-022-01870-3 35759173 PMC9879809

[19] Darif D Hammi I Kihel A El Idrissi Saik I Guessous F Akarid K . The pro-inflammatory cytokines in COVID-19 pathogenesis: What goes wrong? Microb Pathog. (2021) 153:104799. doi: 10.1016/j.micpath.2021.104799 33609650 PMC7889464

[20] Ragab D Salah Eldin H Taeimah M Khattab R Salem R . The COVID-19 cytokine storm; what we know so far. Front Immunol. (2020) 11:1446. doi: 10.3389/fimmu.2020.01446 32612617 PMC7308649

[21] Rajamanickam A Kumar NP Padmapriyadarsini C Nancy A Selvaraj N Karunanithi K . Latent tuberculosis co-infection is associated with heightened levels of humoral, cytokine and acute phase responses in seropositive SARS-CoV-2 infection. J Infect. (2021) 83:339–46. doi: 10.1016/j.jinf.2021.07.029 34329676 PMC8316716

[22] Muema DM Akilimali NA Ndumnego OC Rasehlo SS Durgiah R Ojwach DBA . Association between the cytokine storm, immune cell dynamics, and viral replicative capacity in hyperacute HIV infection. BMC Med. (2020) 18:81. doi: 10.1186/s12916-020-01529-6 32209092 PMC7093991

[23] Huang X Liu X Meyers K Liu L Su B Wang P . Cytokine cascade and networks among MSM HIV seroconverters: implications for early immunotherapy. Sci Rep. (2016) 6:36234. doi: 10.1038/srep36234 27830756 PMC5103227

[24] Roberts L Passmore JAS Williamson C Little F Bebell LM Mlisana K . Plasma cytokine levels during acute HIV-1 infection predict HIV disease progression. AIDS. (2010) 24:819–31. doi: 10.1097/QAD.0b013e3283367836 PMC3001189 20224308

[25] Kleynhans L Maasdorp E Snyders CI Seddon JA . Impact of puberty on immune responses to mycobacterium tuberculosis in south African adolescents (2025) (Accessed March 17, 2026). 10.1093/infdis/jiaf293 PMC12349956 40455886

[26] Herrera M Vera C Keynan Y Rueda ZV . Gaps in study design for immune parameter research for latent tuberculosis infection: A systematic review. J Immunol Res. (2020) 2020:8074183. doi: 10.1155/2020/8074183 32377537 PMC7191376

[27] Kany S Vollrath JT Relja B . Cytokines in inflammatory disease. Int J Mol Sci. (2019) 20:6008. doi: 10.3390/ijms20236008 31795299 PMC6929211

[28] Chi Y Ge Y Wu B Zhang W Wu T Wen T . Serum cytokine and chemokine profile in relation to the severity of coronavirus disease 2019 in China. J Infect Dis. (2020) 222:746–54. doi: 10.1093/infdis/jiaa363 32563194 PMC7337752

[29] Xu ZS Shu T Kang L Wu D Zhou X Liao BW . Temporal profiling of plasma cytokines, chemokines and growth factors from mild, severe and fatal COVID-19 patients. Signal Transduct Target Ther. (2020) 5:100. doi: 10.1038/s41392-020-0211-1 32561706 PMC7303571

[30] Bülow Anderberg S Luther T Berglund M Larsson R Rubertsson S Lipcsey M . Increased levels of plasma cytokines and correlations to organ failure and 30-day mortality in critically ill Covid-19 patients. Cytokine. (2021) 138:155389. doi: 10.1016/j.cyto.2020.155389 33348065 PMC7833204

[31] Lu Q Zhu Z Tan C Zhou H Hu Y Shen G . Changes of serum IL‐10, IL‐1β, IL‐6, MCP‐1, TNF‐α, IP‐10 and IL‐4 in COVID‐19 patients. Int J Clin Pract. (2021) 75:e14462. doi: 10.1111/ijcp.14462 34107113 PMC8237069

[32] Singh S Anshita D Ravichandiran V . MCP-1: Function, regulation, and involvement in disease. In: Int Immunopharmacol, vol. 101. (2021). p. 107598. Available online at: https://linkinghub.elsevier.com/retrieve/pii/S1567576921002344 (Accessed June 30, 2026). 10.1016/j.intimp.2021.107598 PMC8135227 34233864

[33] Slight SR Khader SA . Chemokines shape the immune responses to tuberculosis. In: Cytokine and Growth Factor Reviews, vol. 24. (2013). p. 105–13. doi: 10.1016/j.cytogfr.2012.10.002 PMC3582802 23168132

[34] Luo J Zhang M Yan B Li F Guan S Chang K . Diagnostic performance of plasma cytokine biosignature combination and MCP-1 as individual biomarkers for differentiating stages Mycobacterium tuberculosis infection. J Infect. (2019) 78:281–91. doi: 10.1016/j.jinf.2018.10.017 30528869

[35] Mensah GI Amponsah JA Alahaman NB Anim-Baidoo I Tetteh JKA Addo KK . Performance of eight serum cytokine/chemokine biomarkers in discriminating between active and latent tuberculosis infection in Ghana. Front Immunol. (2025) 16. doi: 10.3389/fimmu.2025.1600712l PMC12179143 40547033

[36] Temu TM Polyak SJ Wanjalla CN Mandela NA Dabee S Mogaka JN . Latent tuberculosis is associated with heightened levels of pro-and anti-inflammatory cytokines among Kenyan men and women living with HIV on long-term antiretroviral therapy. AIDS. (2023) 37:1065–75. doi: 10.1097/QAD.0000000000003523 PMC10155699 36928263

[37] Fisher KL Moodley D Rajkumar-Bhugeloo K Baiyegunhi OO Karim F Ndlovu H . Elevated IP-10 at the protein and gene level associates with pulmonary TB. Front Cell Infect Microbiol. (2022) 12:908144. doi: 10.3389/fcimb.2022.908144 35694534 PMC9184682

[38] Wergeland I Pullar N Assmus J Ueland T Tonby K Feruglio S . IP-10 differentiates between active and latent tuberculosis irrespective of HIV status and declines during therapy. J Infect. (2015) 70:381–91. doi: 10.1016/j.jinf.2014.12.019 25597826

[39] Hong JY Jung GS Kim H Kim YM Lee HJ Cho SN . Efficacy of inducible protein 10 as a biomarker for the diagnosis of tuberculosis. Int J Infect Dis. (2012) 16:e855–9. doi: 10.1016/j.ijid.2012.07.013 22959355

[40] Shang Z Wang J Kuai S Zhang Y Ou Q Pei H . Serum macrophage migration inhibitory factor as a biomarker of active pulmonary tuberculosis. Ann Lab Med. (2018) 38:9–16. doi: 10.3343/alm.2018.38.1.9 29071813 PMC5700157

[41] Wang Q Han W Niu J Sun B Dong W Li G . Prognostic value of serum macrophage migration inhibitory factor levels in pulmonary tuberculosis. Respir Res. (2019) 20:50. doi: 10.1186/s12931-019-1004-3 30841876 PMC6402089

[42] Aksakal A Kerget B Kerget F Aşkın S . Evaluation of the relationship between macrophage migration inhibitory factor level and clinical course in patients with COVID‐19 pneumonia. J Med Virol. (2021) 93:6519–24. doi: 10.1002/jmv.27189 34241898 PMC8426684

[43] Farzad F Yaghoubi N Zahedi Avval F Khadem-Rezaiyan M Jabbari Azad F Youssefi M . Increasing levels of macrophage migration inhibitory factor in COVID-19 infection and its pathophysiological role; cut-off value determination might be clinically misleading. Arch Clin Infect Dis. (2023) 18:e133714. doi: 10.5812/archcid-133714

[44] Bleilevens C Soppert J Hoffmann A Breuer T Bernhagen J Martin L . Macrophage migration inhibitory factor (MIF) plasma concentration in critically ill COVID-19 patients: A prospective observational study. Diagnostics. (2021) 11:332. doi: 10.3390/diagnostics11020332 33671433 PMC7922575

[45] Zivancevic-Simonovic S Minic R Cupurdija V Stanojevic-Pirkovic M Milosevic-Djordjevic O Jakovljevic V . Transforming growth factor beta 1 (TGF-β1) in COVID-19 patients: relation to platelets and association with the disease outcome. Mol Cell Biochem. (2023) 478:2461–71. doi: 10.1007/s11010-023-04674-7 36869188 PMC9984293

[46] Frischbutter S Durek P Witkowski M Angermair S Treskatsch S Maurer M . Serum TGF‐β as a predictive biomarker for severe disease and fatality of COVID‐19. Eur J Immunol. (2023) 53:2350433. doi: 10.1002/eji.202350433 37386908

[47] Dash SP Gupta S Sarangi PP . Monocytes and macrophages: Origin, homing, differentiation, and functionality during inflammation. Heliyon. (2024) 10:e29686. doi: 10.1016/j.heliyon.2024.e29686 38681642 PMC11046129

[48] Yin X Xiao D Yang J . Emerging insights into CC and CXC chemokines and their receptors in Mycobacterium tuberculosis infection. FEBS Open Bio. (2026) 16:1244–58. doi: 10.1002/2211-5463.70269 PMC13327060 42118679

[49] Mvubu NE . Innate immune recognition of Mycobacterium tuberculosis: receptor engagement and inflammatory outcomes at the site of infection. Cell Surf. (2025) 14:100150. doi: 10.1016/j.tcsw.2025.100150 40893408 PMC12396584

[50] Zhang H Cao X Xin H Liu J Pan S Guan L . Serum level of IL-1ra was associated with the treatment of latent tuberculosis infection in a Chinese population. BMC Infect Dis. (2020) 20:330. doi: 10.1186/s12879-020-05047-x 32384874 PMC7206663

[51] Jason J Archibald LK Nwanyanwu OC Byrd MG Kazembe PN Dobbie H . Comparison of serum and cell-specific cytokines in humans. Clin Diagn Lab Immunol. (2001) 8:1097–103. doi: 10.1128/cdli.8.6.1097-1103.2001 PMC96232 11687446

