# Supplementary material for: Comparative serum cytokine and chemokine profiles across tuberculosis, latent TB, HIV, and COVID-19 infections in Namibian adults
Source: Front Immunol. 2026 Oct 2;17:1954262. doi: 10.3389/fimmu.2026.1954262 (PMC13672220; doi:10.3389/fimmu.2026.1954262)
Supplement: Supplementary file 1 [file SupplementaryFile1.docx]

Supplementary Material

# 1 Figure

**Supplementary Figure 1. Flow chart of the study participants.** The study forms part of the project entitled: Collision of three global pandemics: the effect of tuberculosis and HIV on the epidemiological, clinical, virological and immunological trajectory of COVID-19 in Botswana and Namibia (Core-NB), which did the participants’ enrolment. For this study, only participants with known statuses of TB (confirmed by GeneXpert Ultra), LTBI (determined by QuantiFERON-TB Gold Plus test), COVID-19 (determined by qRT-PCR-based SARS-CoV-2), HIV (Rapid test) were included in this study. The control group was made up of individuals who are tested negative for all the four conditions and were considered as control participants. The asterisk (*) indicates participants whose serum samples were excluded from the study due to insufficient sample volume or inability to locate the corresponding stored sample, except for LTBI group. Given the substantially larger number of eligible LTBI participants, only a subset of 81 participants was selected randomly with no pre-specified quota for inclusion in this study.. The double right quotation marks (’’) represents the combined groups: TB mono infection with TB/HIV co-infection and COVID-19 mono-infection with COVID-19/HIV and COVID-19/LTBI co-infection, respectively.

# 2 Tables

| **Supplementary Table I.** Cytokines and chemokines serum concentration of the study participants | | | | | | | | |
| --- | --- | --- | --- | --- | --- | --- | --- | --- |
| **Cytokine**  **/Chemokine** | **Median Concentration [IQR] in pg/mL** | | | | | | | |
|  | **Control participants**  **(n=54)** | **LTBI**  **(n=81)** | **TB**  **(n=13)** | **TB''**  **(n=17)** | **COVID-19/LTBI**  **(n=12)** | **COVID-19''**  **(n=19)** | **HIV**  **(n=23)** | **HIV/LTBI**  **(n=28)** |
| Free active TGF-β1 | 140.28  [99.22-206.45] | 62.45  [30.52-108.56] | 106.99  [71.22–134.88] | 106.99  [74.72-138.32] | 85.28  [61.79–130.2] | 80.65  [62.45-142.96] | 96.43  [56.38-152.32] | 177.2  [99.59-236.06] |
| IL-10 | 4.17  [1.02-6.61] | 4.1  [0.56-6.24] | 1.24  [0.33–6.24] | 6.24  [0.46-7.42] | 4.1  [1.36–6.53] | 6.24  [3-7.42] | 4.1  [0.6-5.17] | 4.1  [0.8-7.37] |
| IL-12p70 | 3.56  [1.78-7.17] | 1.97  [1.5-4.32] | 1.9  [1.33–5.3] | 4.05  [1.9-7.5] | 5.1  [3.1–12.54] | 4.62  [1.95-11.41] | 2.04  [1.75-3.76] | 4.49  [2.04-10.23] |
| IL-1RA | 337.5  [233.19-500.13] | 337.5  [174.78-500.13] | 497.45  [419.3–720.85] | 500.13  [419.3-720.85] | 225.99  [182.36–377.25] | 337.5  [205.49-524.58] | 500.13  [337.5-614.08] | 541.62  [194.2-626.96] |
| IL-8 | 6.67  [3.28-10.14] | 4.83  [2.5-5.87] | 5.28  [5.05–12.29] | 5.28  [4.84-14.75] | 5.05  [3.85–10.8] | 8.64  [4.1-11.64] | 6.67  [3.39-8.17] | 6.7  [6.26-11.22] |
| IP-10 | 144.49  [91.31-190.71] | 139.11  [86.34-186.34] | 475.23  [86.34–1164.93] | 503.45  [86.34-813.35] | 169.34  [122.51–186.34] | 186.34  [133.37-264.53] | 149.35  [127.63-204.38] | 221.51  [146.79-376.65] |
| MCP-1 | 75.56  [50.88-108.71] | 70.27  [40.28-106.63] | 139.61  [111.23–185.77] | 170.09  [117.6-193.71] | 127.27  [73.8–190.69] | 160.65  [71.53-257.42] | 73.27  [62.63-160.65] | 136.13  [97.52-207.6] |
| MIF | 4527.27  [1886.05-8143.86] | 3397.68  [1876.86-5368.58] | 7546.6  [4276.7–11054.95] | 7546.6  [4276.7-11295.6] | 3202.78 [2759.95–6803.33] | 3681.85  [3022.65-7095.05] | 7530.1  [3022.65-14127.2] | 4527.27  [3397.68-9841.76] |
| NCAM | 54771.08  [37498.13-75016.53] | 38202.55  [22745.74-68317.77] | 37549.61 [25240.08–68061.28] | 32481.91  [25240.08-55856.78] | 47097.46 [36981.31–54298.38] | 51941.8  [41182.98-59892.05] | 61294.41  [47359.61-84619.73] | 54011.34  [39056.52-86451.97] |
| sTREM-1 | 65.2  [35.37-92.67] | 35.37  [21.78-73.21] | 35.37  [21.78–44.2] | 36.3  [22.3-68.35] | 27.79  [11.3–68.11] | 35.37  [21.78-83.46] | 66.51  [35.37-78.55] | 65.69  [48.06-85.49] |
| **Note:** Data is presented as Median with interquartile range [IQR] in brackets. Abbreviations: n, number of participants. The double right quotation marks (’’) represents the combined groups: TB mono infection with TB/HIV co-infection and COVID-19 mono-infection with COVID-19/HIV and COVID-19/LTBI co-infection, respectively. | | | | | | | | |

| **Supplementary Table II.** Comparison of serum concentrations of cytokines and chemokines amongst control participants, tuberculosis, and latent tuberculosis infections | | | | |  |
| --- | --- | --- | --- | --- | --- |
| **Cytokine/Chemokine** | **Comparison** | **Raw *p* value** | **adj. *p* value** | **AUC [95%CI]** | **Discrimination** |
| Free active TGF-β1 | Control vs LTBI | **1.21E-09** | **3.62E-09** | 0.809 [0.736-0.883] | **Good** |
| Free active TGF-β1 | Control vs TB | 0.047 | 0.053 | 0.679 [0.512-0.846] | Weak |
| Free active TGF-β1 | LTBI vs TB | 0.053 | 0.053 | 0.668 [0.516-0.820] | Weak |
| IL-10 | Control vs LTBI | 0.172 | 0.516 | 0.569 [0.471-0.667] | Poor |
| IL-10 | Control vs TB | 0.374 | 0.561 | 0.58 [0.385-0.775] | Poor |
| IL-10 | LTBI vs TB | 0.732 | 0.732 | 0.53 [0.338-0.722] | Poor |
| IL-12p70 | Control vs LTBI | **0.024** | 0.072 | 0.615 [0.516-0.714] | Weak |
| IL-12p70 | Control vs TB | 0.295 | 0.442 | 0.595 [0.403-0.786] | Poor |
| IL-12p70 | LTBI vs TB | 0.987 | 0.987 | 0.502 [0.302-0.701] | Poor |
| IL-1RA | Control vs LTBI | 0.204 | 0.204 | 0.436 [0.338-0.535] | Poor |
| IL-1RA | Control vs TB | 0.08 | 0.12 | 0.657 [0.487-0.827] | Weak |
| IL-1RA | LTBI vs TB | **0.01** | **0.029** | 0.722[0.565-0.880] | **Acceptable** |
| IL-8 | Control vs LTBI | **0.000122** | **0.000367** | 0.695 [0.602-0.789] | Weak |
| IL-8 | Control vs TB | 0.787 | 0.787 | 0.475 [0.290-0.660] | Poor |
| IL-8 | LTBI vs TB | **0.019** | **0.028** | **0.703 [0.551-0.855]** | **Acceptable** |
| IP-10 | Control vs LTBI | 0.127 | 0.127 | 0.577 [0.476-0.678] | Poor |
| IP-10 | Control vs TB | **0.042** | 0.063 | 0.682[0.463-0.901] | Weak |
| IP-10 | LTBI vs TB | **0.014** | **0.042** | **0.712 [0.489-0.935]** | **Acceptable** |
| MCP-1 | Control vs LTBI | 0.386 | 0.386 | 0.544 [0.445-0.643] | Poor |
| MCP-1 | Control vs TB | **0.003** | **0.004** | **0.772 [0.640-0.904]** | **Acceptable** |
| MCP-1 | LTBI vs TB | **0.000548** | **0.002** | **0.8 [0.687-0.914]** | **Good** |
| MIF | Control vs LTBI | 0.275 | 0.275 | 0.556 [0.454-0.657] | Poor |
| MIF | Control vs TB | **0.021** | **0.031** | **0.708 [0.574-0.842]** | **Acceptable** |
| MIF | LTBI vs TB | **0.001** | **0.004** | **0.778 [0.664-0.892]** | **Acceptable** |
| NCAM | Control vs LTBI | **0.021** | 0.064 | 0.617 [0.522-0.713] | Weak |
| NCAM | Control vs TB | 0.156 | 0.234 | 0.628 [0.437-0.819] | Weak |
| NCAM | LTBI vs TB | 0.818 | 0.818 | 0.52 [0.336-0.705] | Weak |
| sTREM-1 | Control vs LTBI | 0.151 | 0.226 | 0.573 [0.474-0.671] | Poor |
| sTREM-1 | Control vs TB | 0.111 | 0.226 | 0.643 [0.482-0.805] | Weak |
| sTREM-1 | LTBI vs TB | 0.501 | 0.501 | 0.442 [0.283-0.600] | Poor |
| **Note**: Control participants (Control, *n* = 54), individuals with tuberculosis (TB, *n* = 13), and those with latent tuberculosis infection (LTBI, *n* = 81). To reduce the influence of extreme values, data were winsorized at the 5th and 95th percentiles prior to analysis. Statistical comparisons between groups (Control vs LTBI, Control vs TB, and LTBI vs TB) were performed using the Wilcoxon rank-sum test, with *p*-values adjusted for multiple comparisons using the Benjamini–Hochberg method. Statistical significance was defined as adjusted *p* < 0.05. Receiver operating characteristic (ROC) performance was interpreted according to the area under the curve (AUC) as follows: poor (<0.60), weak (0.60–0.69), acceptable (0.70–0.79), good (0.80–0.89), and excellent (≥0.90). | | | | | |

| **Supplementary Table III.** Comparison of serum concentrations of cytokines and chemokines amongst LTBI, COVID-19/LTBI and HIV/LTBI infections | | | | | |
| --- | --- | --- | --- | --- | --- |
| **Cytokine/**  **Chemokine** | **Comparison** | **Raw *p* value** | **adj. *p* value** | **AUC [95%CI]** | **Discrimination** |
| Free active TGF-β1 | LTBI vs COVID-19/LTBI | 0.099 | 0.099 | 0.649 [0.493-0.804] | Weak |
| Free active TGF-β1 | LTBI vs HIV/LTBI | **2.67E-07** | **8.01E-07** | **0.827 [0.733-0.922]** | **Good** |
| Free active TGF-β1 | COVID-19/LTBI vs HIV/LTBI | **0.04** | 0.06 | **0.708 [0.508-0.909]** | **Acceptable** |
| IL-10 | LTBI vs COVID-19/LTBI | 0.425 | 0.6375 | 0.572 [0.397-0.746] | Poor |
| IL-10 | LTBI vs HIV/LTBI | 0.259 | 0.6375 | 0.571 [0.446-0.697] | Poor |
| IL-10 | COVID-19/LTBI vs HIV/LTBI | 1 | 1 | 0.501 [0.304-0.699] | Poor |
| IL-12p70 | LTBI vs COVID-19/LTBI | **0.002** | **0.003** | **0.774 [0.641-0.907]** | **Acceptable** |
| IL-12p70 | LTBI vs HIV/LTBI | **1.84E-04** | **5.53E-04** | **0.738 [0.632-0.843]** | **Acceptable** |
| IL-12p70 | COVID-19/LTBI vs HIV/LTBI | 0.79 | 0.79 | 0.528 [0.330-0.727] | Poor |
| IL-1RA | LTBI vs COVID-19/LTBI | 0.625 | 0.625 | 0.544 [0.368-0.719] | Poor |
| IL-1RA | LTBI vs HIV/LTBI | **0.004** | **0.012** | **0.683 [0.547-0.818]** | **Weak** |
| IL-1RA | COVID-19/LTBI vs HIV/LTBI | 0.063 | 0.095 | 0.688 [0.510-0.865] | Weak |
| IL-8 | LTBI vs COVID-19/LTBI | 0.133 | 0.2 | 0.635 [0.442-0.827] | Weak |
| IL-8 | LTBI vs HIV/LTBI | **3.16E-06** | **9.48E-06** | **0.796 [0.700-0.891]** | **Acceptable** |
| IL-8 | COVID-19/LTBI vs HIV/LTBI | 0.292 | 0.292 | 0.607 [0.389-0.825] | Weak |
| IP-10 | LTBI vs COVID-19/LTBI | 0.221 | 0.221 | 0.61 [[0.432-0.787] | Weak |
| IP-10 | LTBI vs HIV/LTBI | **7.54E-05** | **2.26E-04** | **0.75 [[0.633-0.868]** | **Acceptable** |
| IP-10 | COVID-19/LTBI vs HIV/LTBI | 0.088 | 0.133 | 0.673 [0.493-0.853] | Weak |
| MCP-1 | LTBI vs COVID-19/LTBI | **0.018** | **0.027** | **0.713 [0.552-0.874]** | **Acceptable** |
| MCP-1 | LTBI vs HIV/LTBI | **3.51E-05** | **1.05E-04** | **0.763 [0.661-0.865]** | **Acceptable** |
| MCP-1 | COVID-19/LTBI vs HIV/LTBI | 0.756 | 0.756 | 0.533 [0.322-0.744] | Poor |
| MIF | LTBI vs COVID-19/LTBI | 0.578 | 0.578 | 0.45 [0.304-0.595] | Poor |
| MIF | LTBI vs HIV/LTBI | **0.029** | 0.088 | 0.639 [0.515-0.762] | Weak |
| MIF | COVID-19/LTBI vs HIV/LTBI | 0.135 | 0.204 | 0.652 [0.471-0.832] | Weak |
| NCAM | LTBI vs COVID-19/LTBI | 0.563 | 0.563 | 0.552 [0.433-0.672] | Poor |
| NCAM | LTBI vs HIV/LTBI | 0.047 | 0.141 | 0.627 [0.510-0.744] | Weak |
| NCAM | COVID-19/LTBI vs HIV/LTBI | 0.434 | 0.563 | 0.58 [0.404-0.756] | Poor |
| sTREM-1 | LTBI vs COVID-19/LTBI | 0.252 | 0.252 | 0.602 [0.428-0.777] | Weak |
| sTREM-1 | LTBI vs HIV/LTBI | 0.087 | 0.130 | 0.608 [0.488-0.729] | Weak |
| sTREM-1 | COVID-19/LTBI vs HIV/LTBI | 0.049 | 0.130 | 0.699 [0.514-0.885] | Weak |
| **Note:** Latent tuberculosis (LTBI, n = 81), coronavirus disease 2019 and LTBI (COVID-19/LTBI, n = 12), and those with human immunodeficiency virus and LTBI infection (HIV/LTBI, n = 28). To reduce the influence of extreme values, data were winsorized at the 5th and 95th percentiles prior to analysis. Statistical comparisons between groups (LTBI vs COVID-19/LTBI, LTBI vs HIV/LTBI, and COVID-19/LTBI vs HIV/LTBI) were performed using the Wilcoxon rank-sum test, with p-values adjusted for multiple comparisons using the Benjamini–Hochberg method. Statistical significance was defined as adjusted (adj.) p < 0.05. Receiver operating characteristic (ROC) performance was interpreted according to the area under the curve (AUC) as follows: poor (<0.60), weak (0.60–0.69), acceptable (0.70–0.79), good (0.80–0.89), and excellent (≥0.90). | | | | | |

| **Supplementary Table IV.** Comparison of serum concentrations of cytokines and chemokines amongst control participants, tuberculosis, and COVID-19 infections | | | | |  |
| --- | --- | --- | --- | --- | --- |
| **Cytokine/Chemokine** | **Comparison** | **Raw *p* value** | **adj. *p* value** | **AUC [95%CI]** | **Discrimination** |
| Free active TGF-β1 | Control vs COVID-19'' | **0.012** | **0.036** | **0.695 [0.547-0.844]** | **Weak** |
| Free active TGF-β1 | Control vs TB'' | **0.029** | **0.044** | **0.677 [0.531-0.823]** | **Weak** |
| Free active TGF-β1 | TB'' vs COVID-19'' | 0.739 | 0.739 | 0.534 [0.338-0.730] | Poor |
| IL-10 | Control vs COVID-19'' | 0.179 | 0.537 | 0.604 [0.454-0.754] | Weak |
| IL-10 | Control vs TB'' | 0.903 | 0.903 | 0.51 [0.333-0.687] | Poor |
| IL-10 | TB'' vs COVID-19'' | 0.362 | 0.543 | 0.59 [0.396-0.783] | Poor |
| IL-12p70 | Control vs COVID-19'' | 0.215 | 0.464 | 0.596 [0.442-0.751] | Poor |
| IL-12p70 | Control vs TB'' | 0.871 | 0.871 | 0.486 [0.313-0.660] | Poor |
| IL-12p70 | TB'' vs COVID-19'' | 0.309 | 0.464 | 0.601 [0.409-0.792] | Weak |
| IL-1RA | Control vs COVID-19'' | 0.96 | 0.96 | 0.504 [0.345-0.664] | Poor |
| IL-1RA | Control vs TB'' | **0.042** | 0.126 | 0.664 [0.512-0.816] | Weak |
| IL-1RA | TB'' vs COVID-19'' | 0.136 | 0.204 | 0.647 [0.460-0.834] | Weak |
| IL-8 | Control vs COVID-19'' | 0.458 | 0.874 | 0.558 [0.398-0.718] | Poor |
| IL-8 | Control vs TB'' | 0.604 | 0.874 | 0.458 [0.288-0.627] | Poor |
| IL-8 | TB'' vs COVID-19'' | 0.874 | 0.874 | 0.483 [0.286-0.680] | Poor |
| IP-10 | Control vs COVID-19'' | 0.218 | 0.218 | 0.596 [0.446-0.745] | Poor |
| IP-10 | Control vs TB'' | **0.011** | **0.032** | **0.706 [0.518-0.894]** | **Acceptable** |
| IP-10 | TB'' vs COVID-19'' | 0.073 | 0.109 | 0.676 [0.470-0.883] | Weak |
| MCP-1 | Control vs COVID-19'' | **0.009** | **0.013** | **0.704 [0.562-0.845]** | **Acceptable** |
| MCP-1 | Control vs TB'' | **0.000165** | **0.000496** | **0.805 [0.692-0.918]** | **Good** |
| MCP-1 | TB'' vs COVID-19'' | 0.8 | 0.8 | 0.526 [0.324-0.729] | Poor |
| MIF | Control vs COVID-19'' | 0.753 | 0.753 | 0.475 [0.337-0.613] | Poor |
| MIF | Control vs TB'' | **0.01** | **0.015** | **0.709 [0.583-0.836]** | **Acceptable** |
| MIF | TB'' vs COVID-19'' | **0.01** | **0.015** | **0.754 [0.593-0.915]** | **Acceptable** |
| NCAM | Control vs COVID-19'' | 0.584 | 0.584 | 0.543 [0.409-0.676] | Poor |
| NCAM | Control vs TB'' | **0.044** | 0.086 | 0.663 [0.503-0.824] | Weak |
| NCAM | TB'' vs COVID-19'' | 0.057 | 0.086 | 0.687 [0.493-0.882] | Weak |
| sTREM-1 | Control vs COVID-19'' | 0.326 | 0.531 | 0.576 [0.423-0.729] | Poor |
| sTREM-1 | Control vs TB'' | 0.354 | 0.531 | 0.575 [0.421-0.729] | Poor |
| sTREM-1 | TB'' vs COVID-19'' | 0.835 | 0.835 | 0.522 [0.328-0.715] | Poor |
| **Note:** Control participants (Control, n = 54), individuals with tuberculosis (TB’’, n = 17), and those with latent tuberculosis infection (COVID-19'', n = 19). The double right quotation marks (’’) represents the combined group TB mono-infection with TB/HIV co-infection, and COVID-19 mono-infection with COVID-19/HIV and COVID-19/LTBI co-infection, respectively. To reduce the influence of extreme values, data were winsorized at the 5th and 95th percentiles prior to analysis. Statistical comparisons between groups (Control vs TB'', Control vs COVID-19'', and TB'' vs COVID-19'') were performed using the Wilcoxon rank-sum test, with p-values adjusted for multiple comparisons using the Benjamini–Hochberg method. Receiver operating characteristic (ROC) performance was interpreted according to the area under the curve (AUC) as follows: poor (<0.60), weak (0.60–0.69), acceptable (0.70–0.79), good (0.80–0.89), and excellent (≥0.90). | | | | | |

| **Supplementary Table V.** Correlations between serum levels of cyto- and chemokines in individuals with TB, LTBI COVID-19/LTBI and HIV/LTBI | | | | | |
| --- | --- | --- | --- | --- | --- |
| **Condition** | **Correlated analytes** | **Spearman’s**  **rho (ρ)** | **Raw p value** | **adj. p value** | **Strength** |
| TB | IL-1RA vs Free active TGF-β1 | 0.3 | 0.320 | 0.948 | Weak |
| TB | IL-8 vs Free active TGF-β1 | 0.06 | 0.850 | 0.948 | Very weak |
| TB | IL-8 vs IL-1RA | 0.66 | 0.015 | 0.222 | Moderate |
| TB | IP-10 vs Free active TGF-β1 | -0.07 | 0.821 | 0.948 | Very weak |
| TB | IP-10 vs IL-1RA | -0.23 | 0.448 | 0.948 | Weak |
| TB | IP-10 vs IL-8 | -0.01 | 0.978 | 0.978 | Very weak |
| TB | IP-10 vs MCP-1 | 0.13 | 0.676 | 0.948 | Very weak |
| TB | IP-10 vs MIF | 0.04 | 0.885 | 0.948 | Very weak |
| TB | MCP-1 vs Free active TGF-β1 | 0.39 | 0.188 | 0.948 | Weak |
| TB | MCP-1 vs IL-1RA | 0.18 | 0.559 | 0.948 | Very weak |
| TB | MCP-1 vs IL-8 | 0.07 | 0.808 | 0.948 | Very weak |
| TB | MCP-1 vs MIF | 0.05 | 0.873 | 0.948 | Very weak |
| TB | MIF vs Free active TGF-β1 | 0.32 | 0.289 | 0.948 | Weak |
| TB | MIF vs IL-1RA | 0.26 | 0.399 | 0.948 | Weak |
| TB | MIF vs IL-8 | 0.22 | 0.473 | 0.948 | Weak |
| LTBI | IL-12p70 vs Free active TGF-β1 | 0.21 | 0.064 | 0.150 | Weak |
| LTBI | IL-1RA vs Free active TGF-β1 | **0.31** | **0.005** | **0.022** | **Weak** |
| LTBI | IL-1RA vs IL-12p70 | 0.15 | 0.179 | 0.376 | Very weak |
| LTBI | IL-8 vs Free active TGF-β1 | **0.33** | **0.003** | **0.014** | **Weak** |
| LTBI | IL-8 vs IL-12p70 | **0.42** | **0.000** | **0.001** | **Moderate** |
| LTBI | IL-8 vs IL-1RA | -0.06 | 0.567 | 0.661 | Very weak |
| LTBI | IP-10 vs Free active TGF-β1 | 0.14 | 0.222 | 0.423 | Very weak |
| LTBI | IP-10 vs IL-12p70 | -0.04 | 0.742 | 0.816 | Very weak |
| LTBI | IP-10 vs IL-1RA | -0.03 | 0.777 | 0.816 | Very weak |
| LTBI | IP-10 vs IL-8 | 0.09 | 0.412 | 0.586 | Very weak |
| LTBI | IP-10 vs MCP-1 | -0.08 | 0.475 | 0.587 | Very weak |
| LTBI | IP-10 vs MIF | -0.09 | 0.442 | 0.586 | Very weak |
| LTBI | MCP-1 vs Free active TGF-β1 | 0.23 | 0.035 | 0.092 | Weak |
| LTBI | MCP-1 vs IL-12p70 | 0 | 0.972 | 0.972 | Very weak |
| LTBI | MCP-1 vs IL-1RA | 0.24 | 0.032 | 0.092 | Weak |
| LTBI | MCP-1 vs IL-8 | -0.12 | 0.277 | 0.447 | Very weak |
| LTBI | MCP-1 vs MIF | **0.33** | **0.003** | **0.014** | **Weak** |
| LTBI | MIF vs Free active TGF-β1 | 0.09 | 0.446 | 0.586 | Very weak |
| LTBI | MIF vs IL-12p70 | **0.27** | **0.014** | **0.049** | **Weak** |
| LTBI | MIF vs IL-1RA | **0.67** | **0.000** | **0.000** | **Moderate** |
| LTBI | MIF vs IL-8 | -0.13 | 0.248 | 0.434 | Very weak |
| COVID-19/LTBI | IL-12p70 vs Free active TGF-β1 | 0.61 | 0.034 | 0.511 | Moderate |
| COVID-19/LTBI | IL-1RA vs Free active TGF-β1 | 0.22 | 0.495 | 0.826 | Weak |
| COVID-19/LTBI | IL-1RA vs IL-12p70 | 0.28 | 0.374 | 0.802 | Weak |
| COVID-19/LTBI | IL-8 vs Free active TGF-β1 | -0.37 | 0.235 | 0.802 | Weak |
| COVID-19/LTBI | IL-8 vs IL-12p70 | -0.25 | 0.438 | 0.822 | Weak |
| COVID-19/LTBI | IL-8 vs IL-1RA | -0.32 | 0.309 | 0.802 | Weak |
| COVID-19/LTBI | IP-10 vs Free active TGF-β1 | -0.02 | 0.947 | 0.974 | Very weak |
| COVID-19/LTBI | IP-10 vs IL-12p70 | 0.15 | 0.634 | 0.865 | Very weak |
| COVID-19/LTBI | IP-10 vs IL-1RA | 0.18 | 0.570 | 0.854 | Very weak |
| COVID-19/LTBI | IP-10 vs IL-8 | -0.06 | 0.843 | 0.974 | Very weak |
| COVID-19/LTBI | IP-10 vs MCP-1 | 0.31 | 0.329 | 0.802 | Weak |
| COVID-19/LTBI | MCP-1 vs Free active TGF-β1 | 0.32 | 0.318 | 0.802 | Weak |
| COVID-19/LTBI | MCP-1 vs IL-12p70 | 0.01 | 0.974 | 0.974 | Very weak |
| COVID-19/LTBI | MCP-1 vs IL-1RA | -0.29 | 0.356 | 0.802 | Weak |
| COVID-19/LTBI | MCP-1 vs IL-8 | -0.01 | 0.974 | 0.974 | Very weak |
| HIV/LTBI | IL-12p70 vs Free active TGF-β1 | -0.07 | 0.715 | 0.825 | Very weak |
| HIV/LTBI | IL-1RA vs Free active TGF-β1 | **0.57** | **0.002** | **0.025** | **Moderate** |
| HIV/LTBI | IL-1RA vs IL-12p70 | 0 | 0.982 | 0.982 | Very weak |
| HIV/LTBI | IL-8 vs Free active TGF-β1 | 0.41 | 0.031 | 0.067 | Moderate |
| HIV/LTBI | IL-8 vs IL-12p70 | 0.41 | 0.030 | 0.067 | Moderate |
| HIV/LTBI | IL-8 vs IL-1RA | **0.49** | **0.009** | **0.043** | **Moderate** |
| HIV/LTBI | IP-10 vs Free active TGF-β1 | **0.45** | **0.015** | **0.046** | **Moderate** |
| HIV/LTBI | IP-10 vs IL-12p70 | 0.33 | 0.087 | 0.164 | Weak |
| HIV/LTBI | IP-10 vs IL-1RA | **0.51** | **0.005** | **0.038** | **Moderate** |
| HIV/LTBI | IP-10 vs IL-8 | 0.22 | 0.258 | 0.388 | Weak |
| HIV/LTBI | IP-10 vs MCP-1 | 0.05 | 0.797 | 0.854 | Very weak |
| HIV/LTBI | MCP-1 vs Free active TGF-β1 | 0.29 | 0.137 | 0.228 | Weak |
| HIV/LTBI | MCP-1 vs IL-12p70 | -0.19 | 0.329 | 0.448 | Very weak |
| HIV/LTBI | MCP-1 vs IL-1RA | **0.46** | **0.013** | **0.046** | **Moderate** |
| HIV/LTBI | MCP-1 vs IL-8 | 0.15 | 0.445 | 0.556 | Very weak |
| Abbreviations: TB, tuberculosis; LTBI, latent TB infection; HIV, human immunodeficiency virus; IL, interleukin; MCP-1, monocyte chemoattractant protein-1; MIF, macrophage migration inhibitory factor; IP-10, interferon gamma-induced protein 10; vs, Versus. Correlation between the analytes were performed using the Spearman’s rank correlation test, with p-values adjusted for multiple comparisons using the Benjamini–Hochberg method. Statistical significance was defined as adjusted (adj.) p < 0.05. Correlation strength for spearman rho was defined as strong ≥ 0.70, moderate (M), 0.40-0.69, weak (W), 0.20-0.39. | | | | | |
